# A Novel Blockade CD47 Antibody With Therapeutic Potential for Cancer

**DOI:** 10.3389/fonc.2020.615534

**Published:** 2021-01-05

**Authors:** Fangzhen Lin, Mengshang Xiong, Wei Hao, Yuewen Song, Ruoqi Liu, Yuanyuan Yang, Xiangfei Yuan, Dongmei Fan, Yizi Zhang, Mu Hao, Zhou Ye, Yang Lu, Yanjun Zhang, Jianxiang Wang, Dongsheng Xiong

**Affiliations:** ^1^ State Key Laboratory of Experimental Hematology, National Clinical Research Center for Blood Diseases, Institute of Hematology & Blood Diseases Hospital, Chinese Academy of Medical Sciences & Peking Union Medical College, Tianjin, China; ^2^ Department of Pharmacy, Tianjin Medical University General Hospital, Tianjin, China; ^3^ Tianjin Institute of Integrative Medicine for Acute Abdominal Diseases, Tianjin Nankai Hospital, Tianjin, China; ^4^ Central Hospital of Karamay, Karamay, Xinjiang, China

**Keywords:** anti-CD47 mAb, CD47, cancer therapy, immunotherapy, phagocytosis

## Abstract

Macrophages as components of the innate immune system play a critical role in antitumor responses. Strategies for targeting CD47 are becoming a hot spot for cancer therapy. The expression of CD47 is exercised by macrophages to make a distinction between “self” or “nonself.” Anti-CD47 antibodies block the interaction between macrophage signal regulatory protein-α (SIRPα) and tumor surface CD47. In this study, we report and assess a novel anti-CD47 blocking antibody named 2C8, which exhibits high affinity and tremendous anticancer effects. More concretely, 2C8 significantly induces macrophages, including protumorigenic subtype M2 macrophages killing tumor cells *in vitro*, and is revealed to be more effective than commercially available anti-CD47 mAb B6H12.2. In vivo, 2C8 controls tumor growth and extends survival of xenograft mice. The antitumor ability of 2C8 might be applicable to many other cancers. The generation of a novel CD47 antibody contributes to consolidating clinical interest in targeting macrophages for the treatment of malignancy and, moreover, as a supplement therapy when patients are resistant or refractory to other checkpoint therapies or relapse after such treatments.

## Introduction

Multiple lines of evidence indicate that immunotherapies, including checkpoint inhibitors such as programmed cell death protein 1 (PD-1) and cytotoxic T-lymphocyte-associated protein 4 (CTLA-4), are efficient strategies to shoot tumor cells (1). Unfortunately, only a minority of patients with certain tumor types can benefit from durable responses from immunotherapies. Particularly for non-Hodgkin’s lymphoma (NHL) and the other solid malignancy patients, little progress has been observed. Poor cell infiltratation and an immunosuppressive tumor milieu are important reasons (2).

Tumor-associated macrophages (TAMs) abundantly infiltrate most solid tumors and represent up to 50% of leukocytes in the tumor microenvironment. Generally, TAMs display an M2-like phenotype (anti-inflammatory function); however, macrophages are heterogeneous and can be modulated by the surrounding microenvironment from an M2- to an M1-like phenotype (pro-inflammatory function) (3). The rate of M2/M1 is associated with a poor prognosis in clinical outcomes, including with NHL (4–8). Given that macrophages are required for chemotherapy and immunotherapy (9, 10), targeting TAMs is designed to reprogram TAMs to a tumoricidal phenotype M1 rather than diminish TAMs directly so that it can reinvigorate antitumor immunity. There are multiple strategies for reorienting TAMs, including the CD47 antibody (11–14). One adequate approach that aims to activate TAMs is blocking CD47 (4, 15, 16).

As an integrin-associated protein, CD47 is ubiquitously expressed on the cell surface (17) and mediates immune escape from macrophage-mediated phagocytosis when it interacts with phagocyte-expressed signal regulatory protein alpha (SIRPα), a protein expressed in macrophages and dendritic cells (18). An increased expression of CD47 has been demonstrated in multiple solid and hematological malignancies compared with normal cells, including but not limited to breast (19), small-cell lung (20), colon (21), ovarian (22), acute myeloid leukemia (AML) (23), and acute lymphocytic leukemia (ALL) (24) malignancies. Targeting the CD47–SIRPα axis promotes macrophage migration into the tumor mass (14), leading to TAMs being functionally switched from a tumor-promoting M2-like phenotype to a tumoricidal M1-like phenotype and enabling TAMs to attack the tumor. In xenograft mouse models, blocking CD47 led to an increased presence of M1-like TAMs (12, 13).

We generated 2C8, a novel monoclonal antihuman CD47 antibody that has displayed high specificity and affinity for CD47 protein and stimulates M0, M1, and M2 macrophage-mediated phagocytosis more effectively in comparison to commercially available anti-CD47 mAb B6H12.2 *in vitro*, suppressing tumor growth *in vivo* and, thus, prolonging mouse survival. Above all, the 2C8 antibody, which harnesses the ability to induce macrophages to eliminate tumor cells, is a promising candidate for cancer therapy.

## Materials and Methods

### Cell Culture

The murine fibroblast cell line 3T3; human embryonic kidney cell–derived 293T cell line; human acute T cell leukemia cell line Jurkat; human chronic myelogenous leukemia cell line K562; human colon cancer cell lines hCT116 and SW620; human leukemia cell line HL60; and human B cell lymphoma cell lines Raji, Daudi, and BJAB were obtained from the Institute of Hematology and Blood Diseases Hospital, Chinese Academy of Medical Science and Peking Union Medical College, Tianjin, China.

### Antibody Generation

293T cells were transfected with pCDH-CMV-MCS-EF1-copGFP-CD47 using X-tremeGENE DNA transfection reagents (Roche) for lentiviral production, and concentration was accomplished using standard protocols. Lentivirus was collected for 3T3 cell infection, and 6–8 h later, lentivirus was removed. After 48 h of infection, CD47 expressing 3T3 (3T3-CD47) cells were established as an immunogen. Six-week-old Balb/c mice were immunized with 3T3-CD47 cells at 2-week intervals for a total of 4 weeks. Blood was collected after immunization by tail bleeding for titer assessment. Hybridomas stably expressing CD47 were generated as standard protocols. In brief, the spleen cells were fused with SP2/0 cells. After the limiting dilution, hybridomas stably expressing CD47 were selected, and supernatants from the resulting clones were screened by flow cytometry analysis. The cDNA of the light (VL) and heavy (VH) variable regions of the 2C8 antibody were obtained by RT-PCR from RNA, which isolated it from the hybridoma.

### Antibody Purification and Characterization

First, 3x10^6^ hybridomas were collected and injected intraperitoneally into 6-week-old Balb/c mice, and 6–10 days later, soluble antibodies in the mouse ascites were purified by protein G HP columns (GE Healthcare) according to the manufacturer’s instructions. Column were washed with PB buffer and eluted protein with the eluting buffer (0.1 M glycine-HCL buffer, pH 3.0). Collected fractions were neutralized with neutralizing buffer (1 M Tris-HCL buffer, pH 9.0). Finally, purified samples were dialyzed against PB buffer. The purity of the eluted antibody fraction was analyzed by sodium dodecyl sulfate polyacrylamide gel electrophoresis (SDS-PAGE) on 12% gels under nonreducing or reducing conditions. Bands were visualized by Coomassie brilliant blue staining. Antibody subtype was detected by Mouse Monoclonal Antibody Isotyping Kit (Roche).

### Antigen Binding Analysis

The 2C8-PE anti-CD47 antibody was generated (China Resources Concord) and diluted into different concentrations to react with the 1x10^6^ CD47 positive cell line Daudi. After 30 min incubation, cells were washed and analyzed by flow cytometry. Kd value was calculated using a nonlinear regression based on the MFI value of PE thereafter, which was performed by GraphPad Prism software.

### Antibody Homology Modeling and Structural Analysis

To model 2C8, we input VH and VL into antibody homology modeling software Discovery Studio. Antibody sequences VH and VL were blasted separately to find the best templates in the protein data bank (PDB), which results in the creation of the homology model of an antibody. 2BRR (PDB ID number), which exhibited 99.1% identity to the 2C8 VL amino acid sequence, and 2ZJS (PDB ID number), revealing 86.6% identity to 2C8 VH amino acid sequence, were chosen for the 2C8 modeling. After CDR loop optimization and energy minimization, the rationality of the modeling structure was assessed by Procheck, Profile-3D, and PROSA. The crystal structure of CD47-ECD (PDB ID: 5TZU) bound to B6H12.2 is publicly available. The binding mode between CD47-ECD and 2C8 was performed by a rigid body docking program ZDOCK. An optimized pose with high ZDOCK score (>12) was typed with the CHARMm Polar H force field and then refined using the RDOCK program. Finally, we chose the binding poses based on both RDOCK scores and protein binding interface.

### Immunofluorescence Staining

Indicated Jurkat cells were fixed in 4% paraformaldehyde for 15 min and blocked with 1% BSA for 30 min at room temperature. Samples were incubated with primary antibodies 2C8 or B6H12.2 overnight at 4°C. Cells were washed three times in PBS and incubated with APC-conjugated antimouse IgG1 secondary antibodies (Bioscience) for 30 min at room temperature. Nuclei were stained with 1 μg/ml DAPI (Sigma) solution. Images were captured by a two-photon laser scanning confocal microscope (OLYMPUS, FV1200 MPE).

### Preparation of BMDM or Human PBMC-Derived Macrophage

Human PBMC-derived macrophages were prepared from human peripheral blood using density gradient separation. PBMC was enriched by adherence to plates for 1 h at 37°C in FBS-free RPMI 1640 medium. Then, nonadherent cells were removed by extensive washing with PBS. Monocytes were cultured in complete RPMI 1640 medium containing 10 ng/ml recombinant human M-CSF (PeproTech) to induce macrophages. Human macrophages were harvested for phagocytosis assay on day 7.

To generate mouse M1 macrophages, peritoneal or bone marrow cells were isolated from female Balb/c mice and cultured with 5 ng/mL recombinant mouse GM-CSF (PeproTech) for 7 days. To generate M0 or M2 macrophages, bone marrow cells were isolated from female Balb/c mice and cultured with 25 ng/mL recombinant mouse M-CSF (PeproTech) for 7 days. On Day 5, M1 polarization was achieved with further treatment on day 5 by 20 ng/mL IFN-γ (PeproTech) stimulation for 1 h, followed by 100 ng/mL LPS (Sigma-Aldrich) for 48 h. M2 polarization was achieved by further treatment with 20 ng/mL IL-4 (PeproTech) and 20 ng/mL IL-13 (PeproTech) for 48 h. Macrophages were arrested for phagocytosis assay on day 7.

### *In Vitro* Phagocytosis Assay

*In vitro* phagocytosis assays were performed as described previously. Briefly, different tumor cells were CFSE (ThermoFisher) labeled according to the manufacturer’s instructions and incubated with human peripheral blood–derived macrophages or mouse macrophages in the presence of different concentrations of CD47 antibody 2C8 or control antibody for 2 h at 37°C. Cells were washed with serum-free media repeatedly and resuspended in 200 μl media. Then, the cells were analyzed by a high-content screening system (PerkinElmer) to determine the phagocytic rate. The number of macrophages analyzed was more than 3000 per well.

### *In Vivo* Antibody Treatment of Human AML Engrafted Mice

All animal studies were performed in accordance with the guidelines under the Animal Ethics Committee of the Institute of Hematology and Hospital of Blood Diseases, Chinese Academy of Medical Sciences and Peking Union Medical College. For the NHL xenograft model, 1 × 10^7^ Raji or BJAB cells were suspended in PBS and injected subcutaneously into the axillary subcutaneous space of Nod/scid mice (female, 5–6 weeks of age, PUMC, China). After 7-10 days of growth, those tumor-bearing mice were given intravenous injection of indicating dose of 2C8 or PBS once every 3 days for three weeks. Mice were observed daily. Tumor volumes were calculated using (height × weight × weight)/2, and the duration of survival was recorded.

## Results

### Generation of CD47 Blocking Antibody

**Figure 1:**
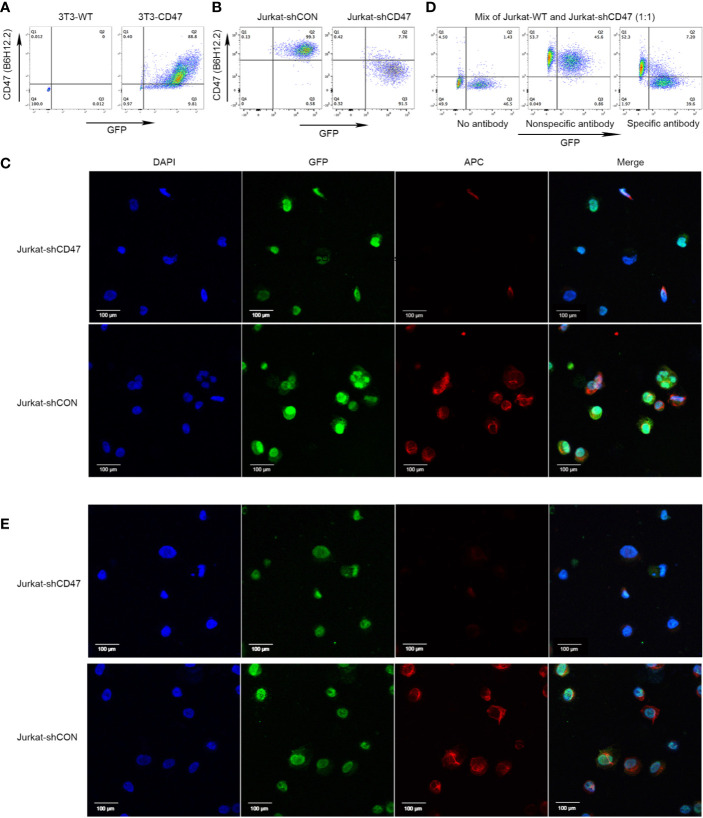
Cloning of antihuman CD47 monoclonal antibodies. **(A)** Representative flow cytometry of 3T3-WT and 3T3-CD47 (GFP) cell line after staining with B6H12.2. **(B)** Representative flow cytometry of Jurkat-shCON (GFP) and Jurkat-shCD47 (GFP) cell line after staining with B6H12.2. **(C)** A pair of Jurkat-GFP cells were stained with B6H12.2 (red) and DAPI (blue) and observed under a two-photon confocal microscope. Scale bar, 100 μm. The experiment was performed three times with similar results. **(D)** A mix of Jurkat-WT cells and Jurkat-shCD47-GFP cells were incubated with hybridoma supernatant and analyzed with flow cytometry. Hybridomas without antibody (left), hybridoma producing nonspecific antibody (middle), or hybridoma producing specific antibody (right). **(E)** A pair of Jurkat-GFP cells were stained with selected hybridoma supernatant (red) and DAPI (blue) and observed under a two-photon confocal microscope. Scale bar, 100 μm. The experiment was performed three times with similar results.

A cDNA fragment of human CD47 encoding the extracellular domain was used to establish CD47 positive cell line 3T3-CD47. Establishment was assessed by flow cytometry using commercially available anti-CD47 mAb B6H12.2 (**Figure 1A**). 3T3-CD47 was used to immunize mice to produce monoclonal mouse antihuman CD47 antibodies. Immunized mice spleen cells were fused with SP2/0 cells. Lentiviral shRNA vectors were used to generate CD47 knockout variants of Jurkat cells, and the efficiency was assessed by flow cytometry (**Figure 1B**). Furthermore, a pair of Jurkat cells were fixed on glass coverslips, incubated with CD47 antibody B6H12.2 overnight at 4°C, and photographed using a confocal microscope and immunofluorescence (**Figure 1C**).

Jurkat-WT cells and Jurkat-shCD47-GFP cells were mixed at a ratio of 1:1 to screen hybridomas that stably expressed CD47 antibodies by flow cytometry analysis. Hybridomas for which supernatants stably reacted with CD47 positive Jurkat cells instead of the CD47 negative Jurkat-shCD47 cell line were selected among three different kinds of hybridomas (**Figure 1D**). In antibody screening, some hybridomas did not produce antibodies (**Figure 1D** left) or produced nonspecific antibodies (**Figure 1D** middle), and we abandoned these hybridomas in subsequent experiments. Finally, nine clones stably producing antihuman CD47 antibodies, specifically binding to CD47 positive tumor cells were obtained. Immunofluorescence further determined clones binding CD47 in a specific manner (**Figure 1E**).

### Characterization of CD47 Blocking Antibody 2C8

**Figure 2:**
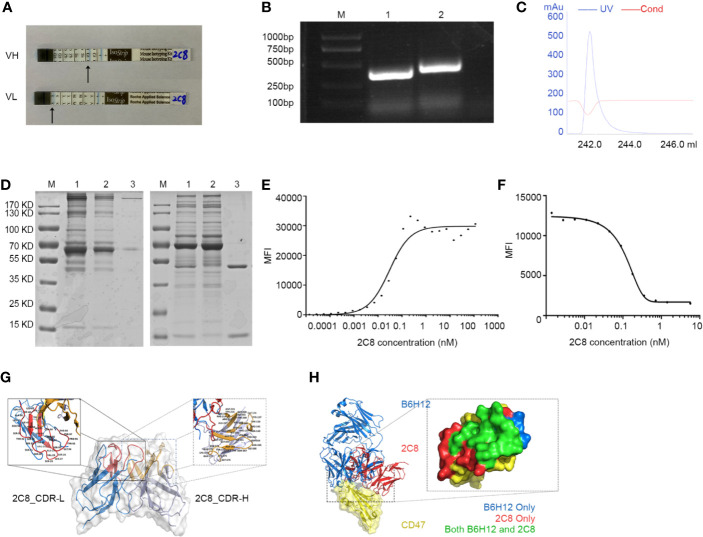
Characterization of antihuman CD47 monoclonal antibody 2C8. **(A)** An antibody subtype was detected by the Mouse Monoclonal Antibody Isotyping Kit. **(B)** VH and VL regions amplified from hybridoma RNA/cDNA. M: marker; Lane 1: 2C8-VL region (330 bp); Lane 2: 2C8-VH region (351 bp). **(C)** UV spectrum of purified 2C8. **(D)** SDS-PAGE was used to show the purified 2C8 antibodies (M: marker, Lane 1, Mouse ascites before purification; Lane 2: purification flow through; Lane 3: purified 2C8). **(E)**. Flow cytometry analysis shows that 2C8 shows a high affinity for CD47, which was 0.2991×10^-9^ M. **(F)** MFI demonstrates that 2C8 disrupts the binding of APC labeled B6H12.2 with Daudi cells. **(G)** Antibody homology modeling structure of 2C8. **(H)** Compound structure of B6H12.2/CD47-ECD superimposed on 2C8/CD47-ECD showing a shared molecular docking. Residues were interacting with only B6H12.2 (blue), only 2C8 (red), or both ligands (green).

After indirect binding reaction screening based on mean fluorescence intensity (MFI) by flow cytometry (data not shown), a clone named 2C8 was chosen for further analysis. The 2C8 antibody subtype was detected by the indicated kit, brands showed that 2C8 was a mouse IgG1 subtype, and the light chain was a kappa chain (**Figure 2A**). RNA was isolated from the 2C8 hybridoma. The cDNA of the VL and VH variable regions of the 2C8 antibody was obtained by RT-PCR using universal antibody primers. VH and VL were successfully cloned, and the corresponding band sizes are shown in **Figure 2B** following purification with a protein G column (**Figure 2C**) and concentrated by centrifugal filters (3K). SDS-PAGE confirmed the purity of 2C8 (**Figure 2D**). The binding assay was carried out *via* flow cytometry analysis. The affinity constant of 2C8-PE was examined by GraphPad software based on MFI value of PE (**Figure 2E**). As expected, 2C8 bound CD47 with a high affinity of 0.2991×10^-9^ M.

A fixed concentration of 2C8 antibodies was mixed with 0.28 nM APC labeled B6H12.2. Mixed antibodies were reactive with Daudi cells. As shown in **Figure 2F**, the MFI value of APC indicated that 2C8 dose-dependently disrupted the interaction between B6H12.2 and CD47. Then, we performed a structural analysis of 2C8 and B6H12.2 in complex with the CD47 extracellular domain. The homology modeling 3-D structure of 2C8 is shown in **Figure 2G**. We simulated the docking of antibodies and antigens and further compared the epitopes recognized by the two antibodies and the amino acid residues involved in antigen binding. As shown in **Figure 2H**, obvious parallels between the 2C8/CD47D-ECD complex and B6H12.2/CD47-ECD complex indicate that 2C8 and B6H12.2 might compete for the same binding site on CD47.

### 2C8 Enables Macrophage-Mediated Phagocytosis

**Figure 3:**
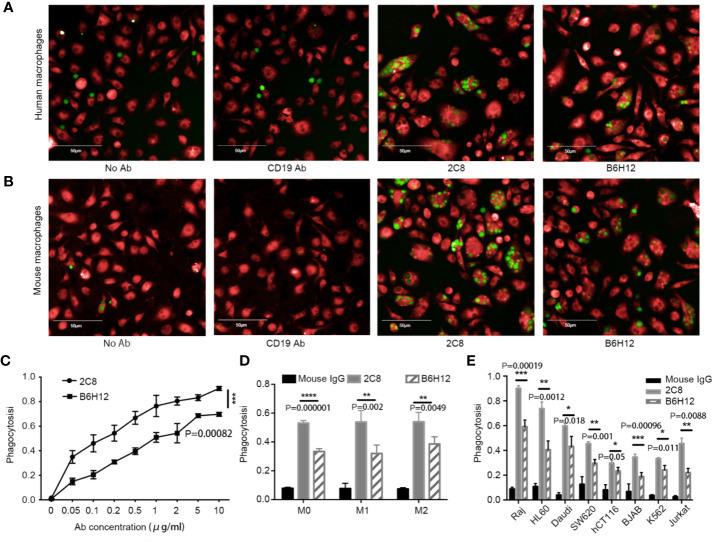
2C8 enables phagocytosis of tumor cells *in vitro*. **(A)** Representative images of human macrophages (red) phagocytosing Raji cells (green) following treatment with the 10 μg/ml indicated antibodies or not. Scale bar, 50 μm. **(B)** Representative images of mouse macrophages (red) phagocytosing Raji cells (green) following treatment with the 10 μg/ml indicated antibodies or not. Scale bar, 50 μm. **(C)** Mouse macrophages phagocytose Raji cells in the presence of 2C8 or B6H12.2 in a concentration-dependent manner. **(D)** Bar graph demonstrating the change of phagocytosis rates by mouse M0, M1, and M2 macrophages toward Raji cells in the presence of 10 μg/ml 2C8 or 10 μg/ml B6H12.2. **(E)** In vitro phagocytosis of multiple tumor cells by mouse macrophages in the presence of 10 μg/ml 2C8 or 10 μg/ml B6H12.2. The data are represented as mean ± SEM. (****p < 0.0001; ***p < 0.001; **p < 0.01; *p < 0.05, multiple *t* tests). The experiment was performed three times with similar results.

Next, we investigated whether 2C8 enabled phagocytosis. The Raji, HL60, Daudi, SW620, hCT116, BJAB, and K562 cell lines were labeled with CFSE (green) and used as target cells; human peripheral blood–derived and mouse macrophages (red) were applied as phagocytes. After 2 h incubation with control antibody CD19 (clone HI19a) or CD47 (2C8 or B6H12.2), phagocytic activity was captured and calculated by a high-content screening system to determine the phagocytic rates. The number of macrophages analyzed was more than 3000 per well. 2C8 treatment induced robust phagocytosis of Raji cells by both human (**Figure 3A**) and mouse macrophages (**Figure 3B** and **Supplementary Video 1**). 2C8 antibodies increased the phagocytosis rate by mouse macrophages in a concentration-dependent manner (**Figure 3C**). We further investigated the phagocytosis function of M1 and M2 subtypes with 2C8 or B6H12.2. The resulting M1 and M2 macrophage phagocytosis rates after 2C8 treatment were statistically significantly increased (**Figure 3D**). We checked the expression of CD47 in Raji, HL60, Daudi, SW620, hCT116, BJAB, and K562 cells (**S1 Fig**). They all expressed a high level of CD47 on the cell surface. Our data indicates that 2C8 increased mouse macrophage-mediated phagocytosis of several types of CD47-positive tumor cells. The ceiling average of phagocytosis rate was more than 90% (**Figure 3E**). These results suggest that 2C8 is a feasible therapeutic agent to eliminate tumor cells. Importantly, 2C8 was more efficacious in comparison to B6H12.2 (**Figures 3C–E**).

### *In Vivo* Antitumor Activity of the 2C8 Antibody

**Figure 4:**
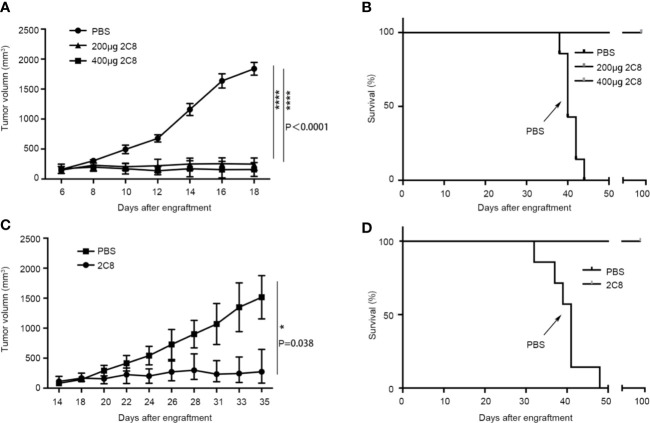
2C8 inhibits tumor growth in xenotransplantation models. **(A)** Raji xenografted mice treated with two different doses of 2C8 or PBS (*n*=7). The tumor volume of Raji tumors per group (*n*=7) is depicted over time. **(B)** Raji xenografted mouse survival after two different doses of 2C8 or PBS treatment (*n* = 7). **(C)** BJAB xenografted mice treated with 200 μg 2C8 or PBS (*n*=4). The tumor volume of BJAB tumors per group (*n*=4) is depicted over time. **(D)** BJAB xenografted mice survival after 200 μg 2C8 or PBS treatment (*n* = 4). The data are represented as mean ± SEM. (****p < 0.0001; *p < 0.05, multiple *t* tests).

We evaluated the antitumor efficiency of 2C8 in NHL xenograft models using NOD/SCID mice to investigate its *in vivo* efficacy using NHL cell line Raji. Consistent with robust phagocytosis induction, as shown in **Figure 4A**, 2C8 was significantly efficacious in controlling Raji tumor growth with low (200 μg/mouse) and high (400 μg/mouse) doses. The final tumor growth inhibition (TGI) values were 91.4% and 86.5%, respectively. 2C8 treatment demonstrated a dramatic increase in survival (**Figure 4B**). We used another NHL cell line BJAB. The final TGI value was 82.0% in controlling BJAB tumor growth with 200 μg/mouse doses. Mice in the control group died within 45 days after tumor cell inoculation, but mice treated with 2C8 demonstrated significantly increased survival (**Figures 4C, D**). These results confirm that 2C8 has potent antitumor activity in a mouse model.

## Discussion

CD47 is highly expressed on multiple tumor cell surface membranes involved in regulating macrophage phagocytosis *via* binding SIRPα to protect host cells from being eliminated. CD47 blocking agents, such as monoclonal antibodies targeting CD47/SIRPα, could interrupt the interaction between cancer cells and macrophages and induce phagocytosis, potentially providing an effective method of cancer therapy. Antitumor mechanisms of the CD47 antibody can be divided into four groups: 1) Enabling phagocytic uptake of tumor cells by macrophages; 2) Promotion of adaptive immunity; 3) Induction of apoptosis; 4) NK cell-mediated ADCC and CDC (25).

The purpose of this study was to describe the generation of a novel blockade antibody 2C8 and assess its biological effects. We first constructed a 2C8 antibody based on the hybridoma technique. An antibody subtype was detected. An akta purification system was used to purify 2C8, and purification was confirmed by SDS-PAGE. Flow cytometry was further performed to assure the binding affinity of 2C8. The higher affinity of 2C8 means a better efficacy for the therapeutic antibodies. 2C8 inhibited APC conjecture B6H12.2 binding with the CD47 positive cell line. Three-dimensional molecular modeling analysis of 2C8 and B6H12.2 in complex with the CD47 extracellular domain suggests that 2C8 and B6H12.2 may partially bind a similar epitope on CD47.

Disruption of the CD47-SIRPα axis by blockade antibody results in enhanced phagocytosis of different kinds of tumor cells, including increased human and mouse macrophage phagocytosis of tumor cells significantly *in vitro* (26). To validate 2C8 as a genuine therapeutic agent, we next performed *in vitro* phagocytosis assays. The data strongly demonstrated that 2C8 was more efficacious than B6H12.2 in promoting phagocytosis. Additionally, the tumor inhibitory role of 2C8 *in vivo* was examined. 2C8 significantly inhibited tumor growth in both Raji and BJAB NHL carcinogenesis models with three times weekly treatment. In addition, 2C8 prolonged the survival of xenografted mice.

In conclusion, 2C8 was effectively generated and possessed ideal attributes. We prepared a CD47 blockade antibody 2C8 to blockade CD47 on tumor cells with high affinity selectively, and it embodied excellent antitumor capability both *in vitro* and *in vivo*. Our prophase results suggest that 2C8 is an efficacious therapeutic agent for human cancer and warrants further studies of 2C8 in different carcinoma diseases. It contributes to consolidate clinical interest in targeting macrophages for the treatment of malignancies and, moreover, as a supplement therapy when patients are resistant or refractory to other checkpoint therapies or relapse after such treatments.

## Data Availability Statement

The original contributions presented in the study are included in the article/**Supplementary Material**. Further inquiries can be directed to the corresponding authors.

## Ethics Statement

The animal study was reviewed and approved by Animal Ethics Committee of the Institute of Hematology & Hospital of Blood Diseases, Chinese Academy of Medical Sciences & Peking Union Medical College.

## Author Contributions

DX, FL, and MX have made substantial contributions to the conception or design of the work. FL, WH, YS, RL, and YY have made substantial contributions to the acquisition of the work. FL, XY, DF, YZZ, and MH have made substantial contributions to the analysis of the work. DX, ZYZ, YL, YJZ, and JW have drafted the work or revised it critically for important intellectual content. All authors contributed to the article and approved the submitted version.

## Funding

This work was supported by the National Natural Science Foundation of China (Grant Nos.81773303, 81830005), Tianjin Municipal Science and Technology Commission Grant (Grant Nos.19JCZDJC65200, 19JCZDJC33100), CAMS Initiative for Innovative Medicine Sciences (CIFMS) (Grant Nos. 2016-I2M-1-007, 2016-I2M-3-013), and Scientific Research Project of Tianjin Education Commission (Grant Nos. 2019KJ196).

## Conflict of Interest

The authors declare that the research was conducted in the absence of any commercial or financial relationships that could be construed as a potential conflict of interest.

## Abbreviations

AML, acute myeloid leukemia; ALL, acute lymphocytic leukemia; CART, chimeric antigen receptor T; CTLA-4, cytotoxic T-lymphocyte-associated protein 4; NHL, non-Hodgkin’s Lymphoma; PD-1, death protein 1; SIRPα, signal regulatory protein-α; SDS-PAGE, sodium dodecyl sulfate polyacrylamide gel electrophoresis; TAMs, tumor-associated macrophages.
