# Supplementary material for: A Novel Blockade CD47 Antibody With Therapeutic Potential for Cancer
Source: Front Oncol. 2021 Jan 5;10:615534. doi: 10.3389/fonc.2020.615534 (PMC7813985; doi:10.3389/fonc.2020.615534)
Supplement: Supplementary file 1 [file Table_1.docx]

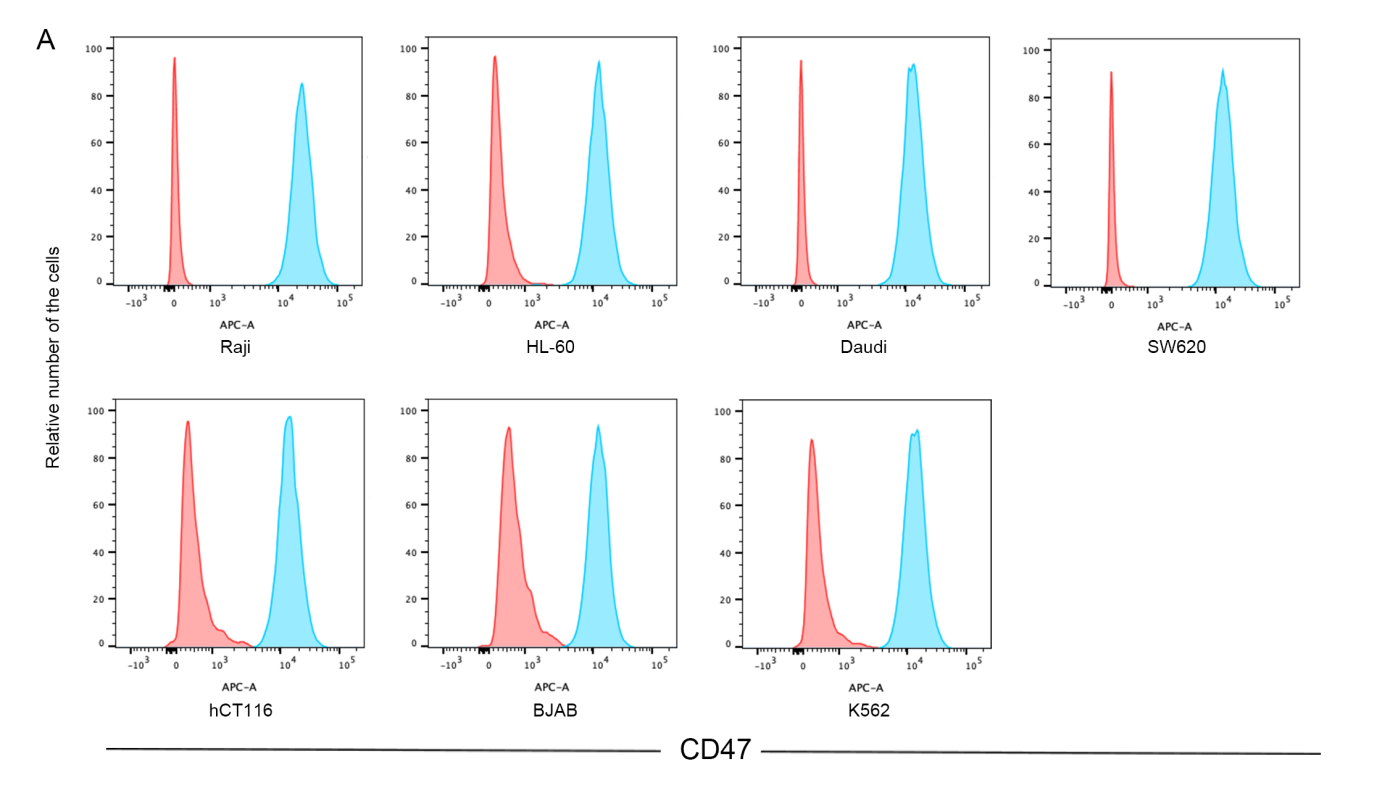


Fig. S1. Cell lines express a high level of cell-surface CD47. (A) Representative plot of surface CD47 expression by flow cytometry. Blue histograms show B6H12-APC stain cells; red histograms are controls.
